# Performance-Enhanced 365 nm UV LEDs with Electrochemically Etched Nanoporous AlGaN Distributed Bragg Reflectors

**DOI:** 10.3390/nano9060862

**Published:** 2019-06-06

**Authors:** Xingdong Lu, Jing Li, Kang Su, Chang Ge, Zhicong Li, Teng Zhan, Guohong Wang, Jinmin Li

**Affiliations:** 1 Semiconductor Lighting Research and Development Center, Institute of Semiconductors, Chinese Academy of Sciences, Beijing 100083, China; luxingdong@semi.ac.cn (X.L.); sukang@semi.ac.cn (K.S.); gechang@semi.ac.cn (C.G.); lizc@semi.ac.cn (Z.L.); zhanteng10@semi.ac.cn (T.Z.); ghwang@semi.ac.cn (G.W.); jmli@red.semi.ac.cn (J.L.); 2 College of Materials Science and Optoelectronic Technology, University of Chinese Academy of Sciences, Beijing 100049, China

**Keywords:** 365 nm UV LED, nanoporous AlGaN DBR, two-step etching, sidewall protection layer, DBR-LED, electrically pumped

## Abstract

A 365-nm UV LED was fabricated based on embedded nanoporous AlGaN distributed Bragg reflectors (DBR) by electrochemical etching. The porous DBR had a reflectance of 93.5% at the central wavelength of 365 nm; this is the highest value of porous AlGaN DBRs below 370 nm which has been reported so far. An innovative two-step etching method with a SiO_2_ sidewall protection layer (SPL) was proposed to protect the n-AlGaN layer and active region of UV LED from being etched by the electrolyte. The DBR-LED with SPL showed 54.3% improvement of maximal external quantum efficiency (EQE) and 65.7% enhancement of optical power at 100 mA without any degeneration in electrical properties, compared with the un-etched standard LED sample. This work has paved the way for the application of electrically-pumped UV LEDs and VCSELs based on nanoporous AlGaN DBRs.

## 1. Introduction

III-nitrides-based near ultraviolet light-emitting diodes (UVA LEDs) have extensive application prospects in ink printing, photoresist curing and nail phototherapy, for they are energy-efficient and mercury-free compared with traditional mercury lamps [1]. One of the most widely used spectra of high-pressure mercury lamps is i-line (365 nm); however, the GaN-based LEDs encounter a significant decrease in efficiency at the emission wavelength shorter than 370 nm [2]. One of the main reasons for the low efficiency is the light absorption from the bottom GaN buffer layers, which have a band gap of 3.43 eV (the absorption edge is 362 nm). The photons emitting downward from the active region are nearly all absorbed by the thick GaN buffer layers and lead to a low light extraction efficiency. A feasible method to avoid the absorption is to insert a “reflector” [3,4] between the GaN buffer layer and the active region, so the downward photons can be reflected back and emit from the top side of LED. In conventional GaN VCSELs, AlN/GaN stacks [5,6,7], AlInN/GaN stacks [8], and AlGaN/GaN stacks [9,10,11,12,13] are grown in situ as the bottom distributed Bragg reflectors (DBRs). However, the Al(Ga)N/GaN systems have encountered a huge challenge of large lattice mismatch, and it is quite difficult to acquire a modest refractive index contrast as well. To create an index contrast of only 0.2 in the AlGaN system would require epitaxy of Al_0.5_Ga_0.5_N on GaN, which is very challenging work. Besides, the epitaxy of Al(Ga)N/GaN stacks is very time-consuming because a large number of periods are required to obtain a high reflectivity. Recently, photoelectronic devices based on nanoporous GaN have been reported [14,15,16,17,18]. By selectively etching the highly Si-doped GaN layers with an electrochemical method, nanoscale air voids are introduced into the n^+^-GaN layer, and thus the refractive index is lowered equivalently. While at the same time, the unintentionally-doped GaN (u-GaN) layers will remain intact, and thus the index contrast is created. The electrochemically-etched u-GaN/n^+^-GaN stack structures have a high refractive index contrast and perfectly matched lattice constant, and high reflectance (>99%) can be obtained in the visible band. Porous AlGaN DBRs have also been reported [19,20] recently in the near UV band, and high reflectance of 90% (at 379 nm) and 93% (at 374 nm) have been obtained, respectively. Yet, almost all the optoelectronic devices based on the porous (Al)GaN DBRs could only be applied in optically pumped conditions so far, because the n-type layers and active regions could also be etched by the electrolytes inevitably, and thus would result in the increase of series resistance and decrease of internal efficiency.

In this work, highly-reflective nanoporous AlGaN DBRs were fabricated by electrochemical anodic oxidation method. The reflectance spectra of nanoporous AlGaN DBRs which were etched under varying bias voltages and in different electrolytes were compared. Then, we proposed an innovative two-step etching method with a SiO_2_ sidewall protection layer (SPL) to isolate the n-type layer and active region of UV LED from the electrolytes. 365-nm UV LEDs based on nanoporous AlGaN DBRs were prepared, and the etching morphology and power enhancement mechanism were analyzed as well. Finally, DBR-LED chips were fabricated and the electroluminescence (EL) spectra, voltage-current (IV) and power-current (PI) characteristics were also analyzed. The light output power of DBR-LED were substantially enhanced without any loss of electrical properties.

## 2. Materials and Methods

All of the samples were grown in C-plane patterned sapphire substrates in a metal organic chemical vapor deposition (MOCVD, AIXTRON, Herzogenrath, Germany) system. Trimethylgallium, trimethylaluminum, trimethylindium, and ammonia were used as gallium, aluminum, indium, and nitrogen sources, respectively. Silane (SiH_4_) and bicyclopentadienylmagnesium (Cp_2_Mg) were n-type and p-type dopants, respectively. Hydrogen and nitrogen were used as carrier gases. The u-AlGaN/n^+^-AlGaN stack structures consist of a 20-nm-thick GaN nucleation layer grown at 580 °C, a 3-μm-thick u-GaN buffer layer grown at 1230 °C, a 500-nm-thick n-Al_5%_GaN (*n* = 1 × 10^18^ cm^−3^) as the current spreading layer, and 15.5 pairs of u-Al_9%_GaN/n^+^-Al_9%_GaN stacks (35 nm/42 nm, *n* = 6 × 10^18^ cm^−3^) grown at 1250 °C. Based on the u-AlGaN/n^+^-AlGaN stack structures, the 365 nm DBR UV LEDs consist of a-500 nm-thick u-Al_5%_GaN as current blocking layer, a 1-μm-thick n-Al_4%_GaN layer (*n* = 8 × 10^18^ cm^−3^), 15 pairs of AlInGaN multiple quantum wells (MQWs, including 12 pairs of Si-doped pre-wells and 3 pairs of undoped MQWs), a 10-nm-thick p-Al_20%_GaN electron-blocking layer, a 80-nm-thick p-Al_6%_GaN layer, and a 20-nm-thick p-GaN cap layer altogether. In the electrochemical etching procedure of the porous AlGaN DBRs, firstly a 1-μm-thick SiO_2_ was deposited on the wafers. Then a series of grooves with 24 μm width and 400 μm spacing were defined by standard photolithography and inductively-coupled plasma (ICP, NAURA, Beijing, China) etching methods. These Grooves were paralleled to (11$\bar{2}$0) planes of AlGaN epilayers (also perpendicular to the locating edge of sapphire substrates) and were etched until the n-Al_5%_GaN conducting layer was exposed. Then the wafers were etched in the electrolyte solution, with the anode of the electrolytic cell contacting with the n-Al_5%_GaN current spreading layer, while the cathode immersed into the electrolyte. We have used the 68% weight ratio nitric acid (HNO_3_) and 1 mol/L potassium hydroxide (KOH) solution as the electrolytes, respectively. The u-AlGaN/n^+^-AlGaN stacks were electrolyzed at different bias voltages under a steady voltage source (Keithley B2400, Cleveland, OH, USA) for about 4–5 h until the etching was completed. After the electrochemical etching process was over, the SiO_2_ was removed by diluted hydrofluoric acid, then the wafers were cleaned with deionized water and dried by nitrogen. The optical microscope images were observed on the wafer surfaces, and the cross-sectional SEM images were observed along the etching direction (also perpendicular to the exposed (11$\bar{2}$0) planes).

In the process of fabricating UV LEDs, a two-step etching method was introduced with a SiO_2_ sidewall protection layer. After electrochemical porosification, the LED chips were fabricated with a standard chip process. First, the mesa was defined by ICP etching using Cl_2_/BCl_3_ gases, and the n-Al_4%_GaN was partly exposed as the n-type layer for the deposition of the metal electrode. Then, a 30-nm-thick transparent conducting layer (ITO) was deposited on the p-GaN, and the ITO was annealed in nitrogen atmosphere at 600 °C for 4 min for better crystalline quality and higher transmittance [21]. The resistivity of ITO after annealing was about 2.7 × 10^−4^ Ω·cm, and the transmittance was about 91.1% at 365 nm, respectively. Next, Cr/Pt/Au (5 nm/500 nm/1000 nm) metals were deposited by E-beam evaporation as the n-type and p-type electrodes. Finally, the wafers were diced into chips of 450 × 750 μm^2^ by laser scribing.

The pore morphologies of the porous AlGaN DBR were characterized by scanning electron microscopy (Hitachi, S-4800, Tokyo, Japan). The light reflectance spectra were measured by UV-Vis-NIR spectrophotometer (Varian Cary-5000, Santa Clara, CA, USA). The standard reference sample we used in the reflectance spectra measurement was a wide-band dielectric mirror (R > 99.5%, 350–450 nm) from Thorlabs company (Newton, NJ, USA), because the conventional reference sample (white board) has a little bit of absorption of UV light and will result in a higher measured reflectance. The photoluminescence (PL) spectra of UV LEDs were measured by μ-PL equipment (Horiba, Kyoto, Japan) with a spot size of less than 5 × 5 μm^2^ using a 325-nm He-Cd laser. The electric field distribution spectra of porous DBR and DBR-LED were simulated by finite-different time-domain (FDTD) method. The EL spectra, IV curves and PI curves were measured by an ultraviolet integrating sphere (200–400 nm, HAAS 2000, Everfine, Hangzhou, China) at room temperature in continuous wave (CW) conditions. The far field distribution patterns were obtained by an angle-resolved spectrum system (R1, Ideaoptics, Shanghai, China).

## 3. Results and Discussion

**Figure 1:**
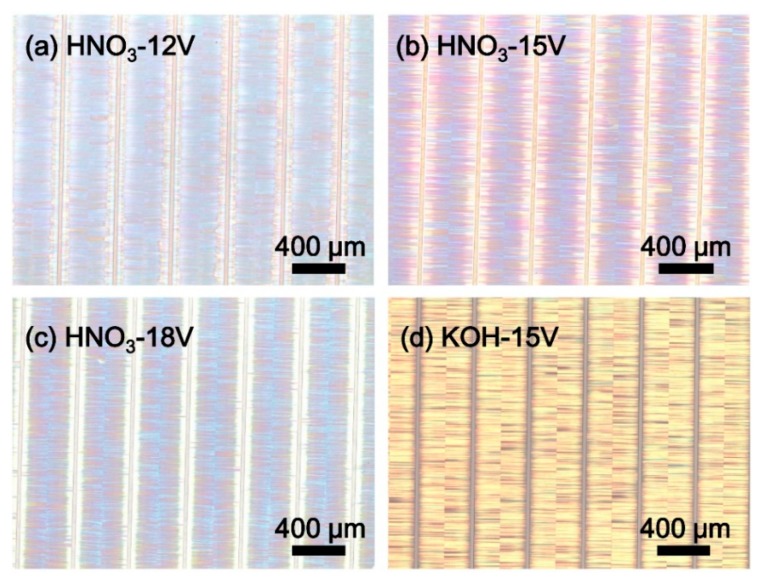
OM-images of porous AlGaN stacks etched in: (**a**) 15 M HNO_3_ at 12 V; (**b**) 15 M HNO_3_ at 15 V; (**c**) 15 M HNO_3_ at 18 V; (**d**) 1 M KOH at 15 V.

Figure 1 shows the optical microscope (OM) images of porous AlGaN stacks etched in different solutions at varying bias voltages. The wafers were divided into a series of rectangular stripes with a 400 μm width and 24 μm spacing (namely, 24 μm groove width). The AlGaN stacks were etched from both sides of the stripe edges, and the etching tunnels were perpendicular to the grooves and eventually converged in the middle of the stripes. The colors of the sample surfaces revealed the different reflective properties when they were electrochemically-etched in different conditions.

**Figure 2:**
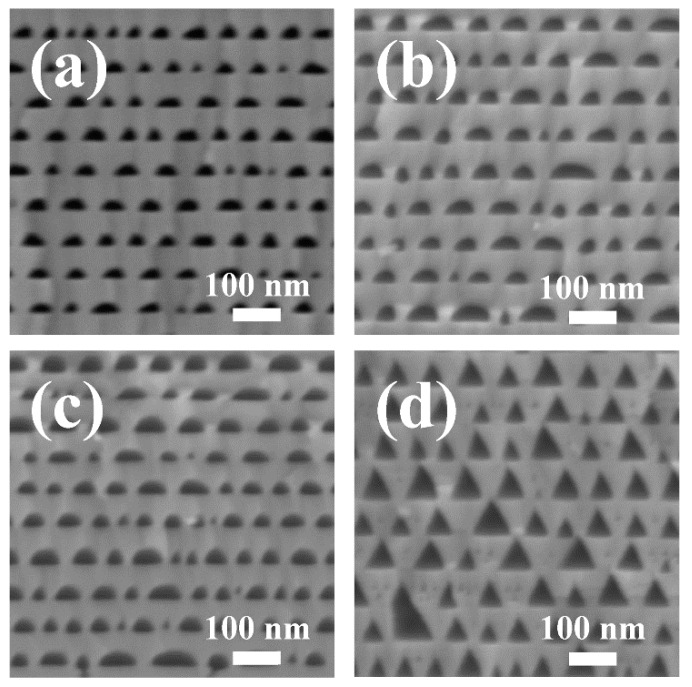
Cross-sectional SEM images of porous AlGaN stacks etched in: (**a**) 15 M HNO_3_ at 12 V; (**b**) 15 M HNO_3_ at 15 V; (**c**) 15 M HNO_3_ at 18 V; (**d**) 1 M KOH at 15 V.

Figure 2 shows the morphologies of porous AlGaN stacks etched in 65% nitric acid at different bias voltages of 12, 15 and 18 V and etched in 1 M KOH solution at 15 V, respectively. The observed planes were (11$\bar{2}$0) crystal planes. From the cross-sectional SEM images of Figure 2a–c, we can find that periodic nanoporous n^+^-AlGaN/un-etched u-AlGaN stacks were obtained by electrochemical (EC) etching in HNO_3_ solution. The n^+^-AlGaN layers were selectively etched in nitric acid, and the porosity rises with the increase of bias voltages. The nano-pores in n^+^-AlGaN layer had a shape between triangle and arch, which should be attributed to the isotropic etching of HNO_3_ to n-AlGaN at high applied voltages, because the crystal planes become unstable and gradually disappear when the oxidation capacity of the anode becomes so strong with the increasing bias voltages [22]. The electrochemical etching mechanism of Ⅲ-nitrides has been reported before [22,23]. It is generally believed that the etching process is mainly controlled by the anode oxidation of the semiconductors and the chemical dissolution of the oxidation products. When a bias voltage was applied to n-AlGaN, the holes would transport to the electrolyte while oxidation happened to n-AlGaN in the AlGaN/HNO_3_ interface:

(1) $${\mathrm{Al}}_{x}{\mathrm{Ga}}_{1-x}N+3H_{2}O+3h^{+}\to x\mathrm{Al}{\left(\mathrm{OH}\right)}_{3}+\left(1-x\right)\mathrm{Ga}{\left(\mathrm{OH}\right)}_{3}+\frac{1}{2}N_{2}+3H^{+}$$

and the thermodynamically unstable oxidization products would dissolve in the electrolyte solution, become Ga^3+^ and Al^3+^:

(2) $$x\mathrm{Al}{\left(\mathrm{OH}\right)}_{3}+\left(1-x\right)\mathrm{Ga}{\left(\mathrm{OH}\right)}_{3}+3H^{+}\to {\mathrm{Ga}}^{3+}+{\mathrm{Al}}^{3+}+3H_{2}O$$

However, when the u-AlGaN/n^+^-AlGaN stacks were etched in 1 M KOH solution, the morphologies of the nano-pores become totally different from the sample etched in HNO_3_ solution, as shown in Figure 2d. The isosceles triangular pore morphology was obtained by EC etching in KOH solution at 15 V voltage. What is more, the pores even stretched into the u-AlGaN layers. It suggests that KOH solution does not only etch the n-doped AlGaN but also reacts with the u-AlGaN. This phenomenon has never been observed before in the EC etching process of porous GaN. We proposed that the etching mechanism of u-AlGaN in KOH solution was mainly controlled by the selective corrosivity of OH^−^ to Al atoms in the N-polar AlGaN surfaces [24]. The typical angle between the pore wall and the bottom N plane was about 63° in Figure 2d, and the reason should be attributed to the exposure of {10$\bar{1}\bar{1}$} family of planes, which usually have a smaller surface energy [23]. The results shown in Figure 2a–d indicated that the etching process of AlGaN in HNO_3_ was mainly controlled by the n-type doping concentration, while in KOH solution it was seriously influenced by the high corrosivity of N-polar surfaces of AlGaN, thus the periodicity of porous AlGaN stacks was severely damaged. Apparently, if we want to obtain a highly reflective nanoporous AlGaN DBR by EC etching, HNO_3_ solution should be a better choice of the electrolyte than KOH solution, for a terrific periodicity of AlGaN stacks could be obtained by the former.

As is known, a distributed Bragg reflector (DBR) consists of a number of quarter-wave high refractive layers and low refractive layers which are piled up periodically. The refractive index and thickness of each layer satisfy the equation of:

(3) $$n_{H}d_{H}=n_{L}d_{L}=\frac{\lambda }{4}$$

where λ is the central wavelength, $n_{H}$ and $d_{H}$ are the index and thickness value of the high index layer, while $n_{L}$ and $d_{L}$ are the index and thickness value of the low index layer, respectively. In the experiment, u-AlGaN is the high index layer, and porous n-AlGaN is the low index layer thanks to the porosification by EC etching. The refractive index of u-Al_9%_GaN is about 2.6 at 365 nm [25], while the index of the porous n^+^-AlGaN layer could be calculated by the volume average theory (VAT) [15,26]:

(4) $$n_{\mathrm{eff}}=\sqrt{\phi n_{\mathrm{air}}^{2}+\left(1-\phi \right)n_{s}^{2}}$$

where the $n_{\mathrm{eff}}$ is the effective index of porous AlGaN layer, ϕ is the porosity, $n_{\mathrm{air}}$ is the index of air, and $n_{s}$ is the index of u-AlGaN, respectively. In fact, we can change the $n_{\mathrm{eff}}$ by varying the applied voltages of EC etching, since the porosity ϕ is closely related to the bias voltage [23,27]. In this work, the porosity ϕ was designed to be 35.6%, and the $n_{\mathrm{eff}}$ was supposed to be 2.17. The index contrast was 0.43 accordingly, and it is sufficient to constitute highly reflective DBRs.

**Figure 3:**
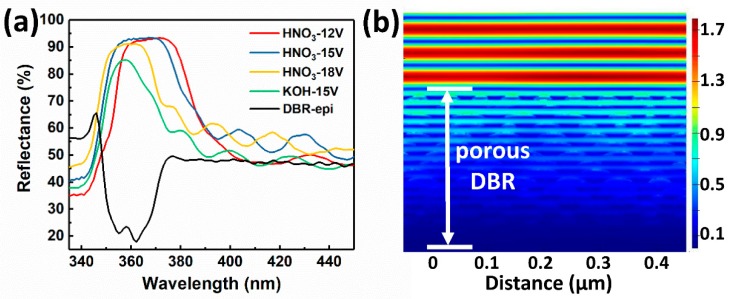
(**a**) The reflectance spectra of porous AlGaN DBRs etched in HNO_3_ and KOH solutions. (**b**) Simulated electric field distribution of porous DBR (HNO_3_-15 V) by FDTD.

The reflectance spectra of porous AlGaN DBRs are shown in Figure 3a. A peak reflectance of 93.5% (20 nm bandwidth) at 365 nm central wavelength was obtained by EC etching in HNO_3_ under 15 V bias voltage. This is the highest reflectance achieved by porous AlGaN DBRs below 370 nm so far. It should be noted that only 15.5 pairs of u-Al/n^+^-Al stacks were used. Nevertheless, the porous AlGaN DBR by EC etching could reach a relatively high reflectance which must be obtained by a number of periods (typically > 40) in conventional AlGaN/GaN DBRs grown in situ [12,13]. When the applied voltage increased from 12 to 18 V (in HNO_3_), the reflectance spectrum shifted to the short wavelength side, due to the decreasing refractive index of porous n^+^-AlGaN layers (or decreasing value of $n_{L}d_{L}$). The reflectance spectrum of DBR etched at 18 V showed a narrowing bandwidth and declining peak value compared with the other two, because it is too close to the band edge of Al_9%_GaN (344 nm) and thus the absorption from u-AlGaN/n^+^-AlGaN stacks cannot be ignored any more. Besides, the DBR etched in KOH solution had the narrowest bandwidth and the lowest reflectance, because the periodicity of u-AlGaN/n^+^-AlGaN stacks was severely destroyed by the etching of OH^−^ to u-AlGaN as shown in Figure 2d. Figure 3b shows the simulated electric field distribution by FDTD method. The cross-sectional SEM image of porous DBR (HNO_3_-15 V) was directly imported as the simulation structure. A plane wave with a wavelength of 365 nm was set as the light source, and the boundary conditions were set as “Periodic” and “Perfect Matching Layers” for the horizontal and vertical direction, respectively. Standing waves were formed above the DBR surface which indicated the good reflection of the quarter-wave porous u-AlGaN/n-AlGaN stacks. The electric field intensity mainly focused on the top layers of AlGaN stacks, which also indicated that the number of u-AlGaN/n-AlGaN pairs could be further reduced. These results suggested that a highly-reflective porous AlGaN DBR in the UV range could be obtained by carefully adjusting the thickness and doping concentration of the un-doped/Si-doped AlGaN layers. The central wavelength of the spectrum could be easily shifted (to a certain extent) just by changing the bias voltages. Compared with the conventional in-situ Al(Ga)N/GaN DBRs, the DBRs fabricated by EC etching could easily reach a high reflectance (>93% in our work), while nearly having no lattice mismatch and the pairs required to obtain the same reflectance were also dramatically reduced due to the large index contrast. Thus, EC etching is a very promising way to replace conventional in-situ epitaxy of Al(Ga)N/GaN in fabricating highly-reflective DBRs.

**Figure 4:**
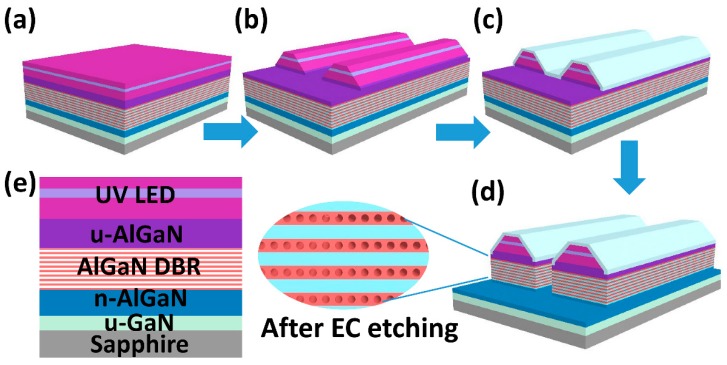
(**a**–**d**) Schematic diagrams of the two-step etching method with a SiO_2_ SPL; (**e**) the epitaxial structure of the composite UV LED with embedded AlGaN DBR.

Now that the high reflective porous AlGaN DBRs were achieved, another problem was how to combine the UV LEDs with the DBR structure together. One way to realize the composite LED structure was performing secondary epitaxy on the nanoporous AlGaN DBRs. However, the nano-pores would transform and merge with each other under a high growth temperature [15]. So the other way was to grow the complete structures (including top LED and bottom AlGaN stacks) altogether by MOCVD, then etching the whole structure with the electrochemical method to get embedded nanoporous AlGaN DBRs. However, thanks to the high selectivity of EC etching to the Si-doped AlGaN, the n-AlGaN layer of UV LED would also be porosified inevitably, thus leading to poor electrical properties and low luminous efficiency. In order to isolate the LED epilayers from electrolyte solution, we proposed an innovative two-step etching method by ICP with a SiO_2_ sidewall protection layer, and the details are shown in Figure 4a–d. First, the composite LED structures based on the u-Al/n^+^-Al stacks were grown in MOCVD and the wafers were cleaned before the experiment. Second, photolithography followed by ICP etching were performed to define the grooves (not mesa) with 24 μm width and 400 μm spacing, and the etching depth was 1.5 μm until the u-AlGaN was exposed. The wafers used here were split into quarters of 2 inches for easier operation of EC etching. It should be noted that the sidewalls were designed to have a sloping angle for better adhesion of SiO_2_, as shown in Figure 4b. The sloping sidewalls were acquired by carefully controlling the exposure time and baking temperature of the photoresist before ICP etching. Next, 1-μm-thick SiO_2_ was deposited by a plasma-enhanced chemical vapor deposition (PECVD, NAURA, Beijing, China) system on the wafer surface, and now the sidewalls of UV LEDs were covered by a compact SiO_2_ layer, as shown in Figure 4c. Then, another photolithography was performed to expose the groove surfaces, followed by wet etching (by diluted hydrofluoric acid solution) to remove the SiO_2_; second, ICP etching was performed successively to expose the sidewalls of AlGaN DBRs, as shown in Figure 4d. At the same time, the bottom n-AlGaN layer was also exposed to serve as the contact layer with the anode of the electrolytic cell. After that, the wafers were carried out by electrochemical etching in HNO_3_ solution to obtain porous AlGaN DBRs. One should be aware that the EC etching direction was perpendicular to the grooves, because the wafer surfaces were well covered by a thick SiO_2_ layer, and thus the vertical etching from p-GaN surface could not happen. When the EC etching was over, the SiO_2_ was removed and the wafers were cleaned by deionized water and dried by nitrogen.

**Figure 5:**
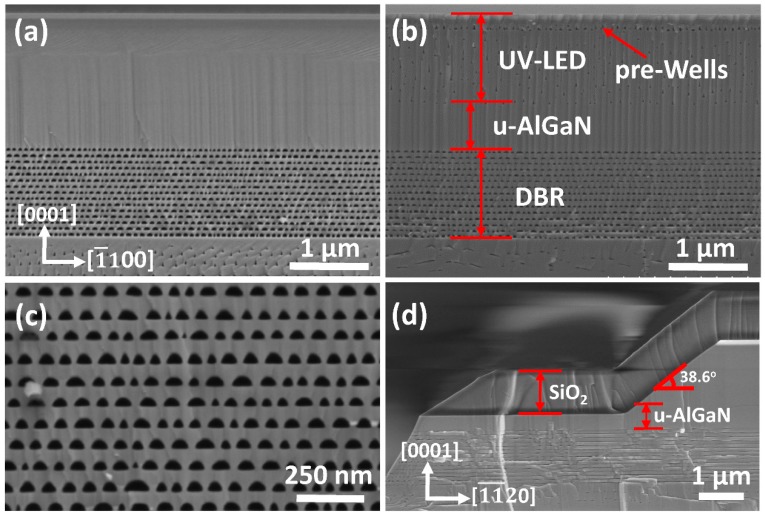
Cross-sectional SEM images of (**a**) DBR-LED with SPL; (**b**) DBR-LED without SPL; (**c**) detailed view of DBR structure in (**a**); (**d**) detailed view of SPL structure in (**a**). The observed crystal planes were (11$\bar{2}$0) planes in (**a**–**c**), and ($\bar{1}$100) plane in (**d**).

The cross-sectional SEM images of DBR-LEDs are shown in Figure 5. Not only the terrific periodic porous n^+^-AlGaN/u-AlGaN stacks were acquired, but also the n-AlGaN and active region of UV LED were still intact after EC etching, as shown in Figure 5a,c. In contrast, the n-AlGaN layer and pre-wells of the DBR-LED without SPL were seriously damaged after EC etching even with a thick u-AlGaN blocking layer, as shown in Figure 5b. The reason for the intact epilayers of DBR-LED in Figure 5a was due to the isolation of electrolyte by the compact SiO_2_ SPL, which typically had a sloping angle of 38.6° as shown in Figure 5d. The sloping sidewall was beneficial for the deposition and adhesion of SiO_2_. The SiO_2_ layer would fall off and SPL would be invalid if it was deposited on a vertical sidewall. The etching trenches were along the [$\bar{1}\bar{1}2$0] direction, and the effect of the u-AlGaN blocking layer was to prevent the trenches climbing upwards to the n-AlGaN layer of UV LED. The results shown in Figure 5 indicated that porous DBR-LEDs were successfully fabricated by EC etching and that SiO_2_ SPL played an important role in the EC process.

**Figure 6:**
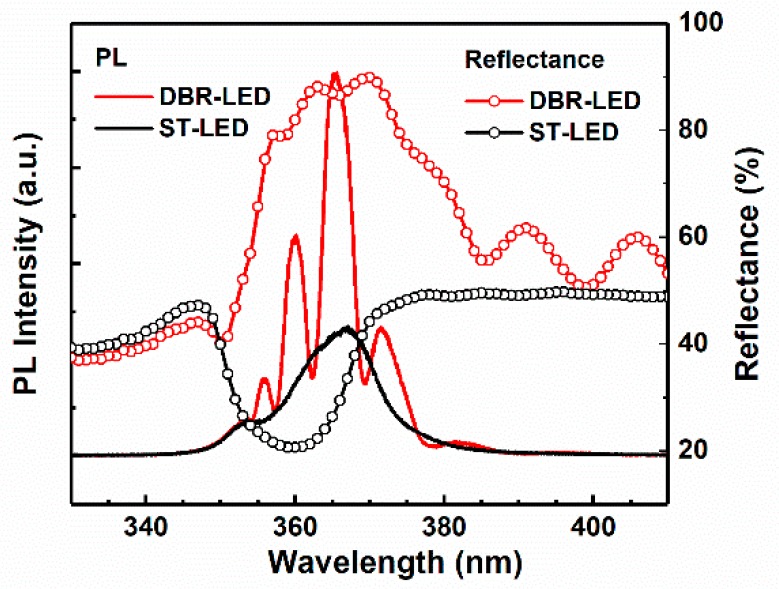
PL spectra and reflectance spectra of DBR-LED and ST-LED.

Figure 6 shows the PL and reflectance spectra of DBR-LED and standard-LED without EC etching (ST-LED). The peak wavelength of DBR-LED and ST-LED were 366.9 and 365.5 nm, respectively. The slight blue-shift of the peak wavelength might be the result of the relaxation of compressive strain in the AlInGaN MQWs [28] due to the porosification of the AlGaN DBR structure. The PL emission intensity of DBR-LED was substantially enhanced by inserting a highly-reflective porous AlGaN DBR, compared with the non-etching ST-LED. While at the same time, a number of secondary emission peaks of DBR-LED were found at 355.8, 360, and 371.6 nm. The appearance of these peaks should be attributed to the resonance effect between the bottom AlGaN DBR and the air/GaN interface. The reflectance of DBR-LED showed a slight decrease compared with the DBR-only structures, which should be the result of the absorption of p-GaN and MQWs in the top UV LED structure. What is more, the concave reflectance spectrum at 366 nm of DBR-LED also indicated the occurrence of resonance phenomenon.

**Figure 7:**
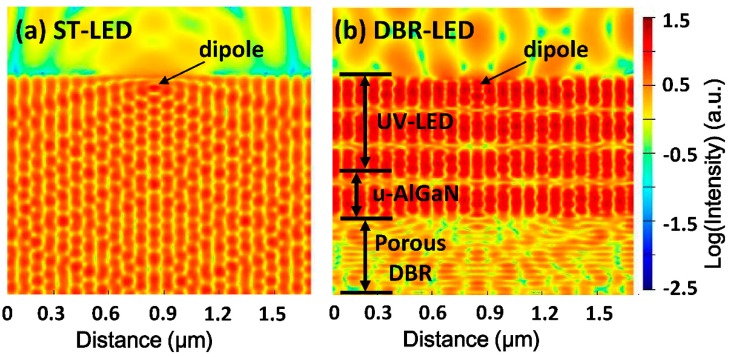
Simulated electric field distribution of (**a**) ST-LED and (**b**) DBR-LED. The light source was a TE polarized dipole, and the electric field intensity is presented in log scale.

Figure 7 shows the simulation results of the electric field intensity distribution of ST-LED and DBR-LED by FDTD. The light source employed in the simulation was a TE polarized dipole (*λ* = 365 nm) in the in-plane direction. The dipole was put in the MQWs region of UV LED, and the refractive index of the UV LED structure was fixed at 2.60 to simplify the simulation. In the ST-LED, the electric field had a uniform distribution inside and only a small amount of photons escaped from the LED surface because of the total internal reflection. In the DBR-LED, the electric field intensity was enhanced in the UV-LED region and reduced in the DBR region, due to the high reflection of bottom porous DBR. The amount of emitting photons were also dramatically increased, which indicated the DBR LED would have a higher light extraction efficiency than the ST-LED.

**Figure 8:**
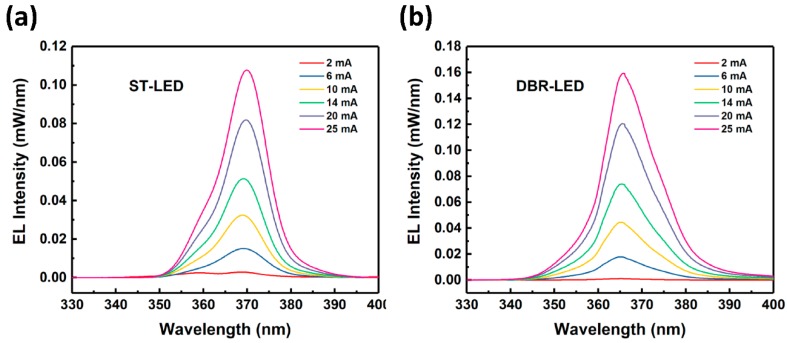
EL spectra of (**a**) ST-LED; and (**b**) DBR-LED at the current from 2 to 25 mA.

Finally, DBR-LED chips were fabricated by standard LED chip technology after EC etching. The EL spectra of DBR-LED and ST-LED chips are shown in Figure 8. The resonance of effect of DBR-LED disappeared due to the damaged air/p-GaN interface during the chip-fabricating process. The EL wavelength was 369.6 and 365.4 nm at 20 mA for ST-LED and DBR-LED, respectively. The blue shift of wavelength could be the result of the relaxation of compressive stress in the AlInGaN MQWs, due to the porosification of AlGaN DBRs, which in turn resulted in the decrease of the quantum-confined Stark effect (QCSE). The EL intensity of DBR-LED was also higher than the ST-LED due to the effect of the highly-reflective porous AlGaN DBR.

**Figure 9:**
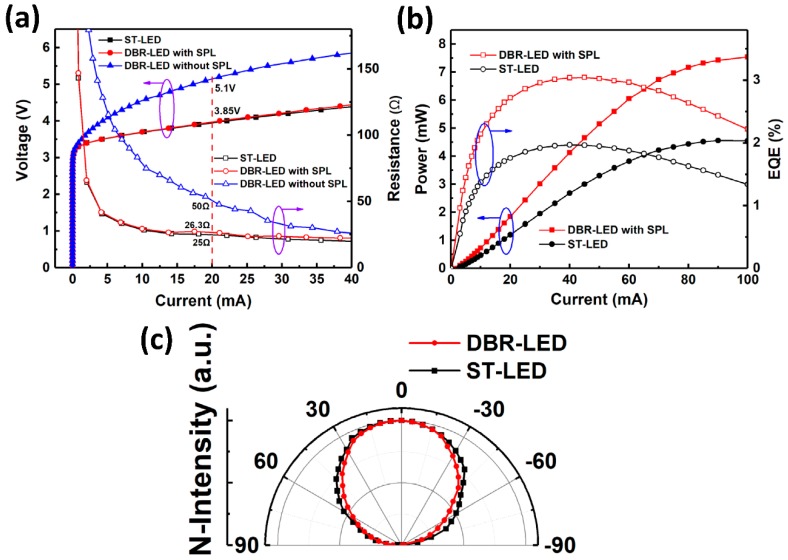
(**a**) I-V characteristics of UV LEDs; (**b**) P-I characteristics of UV LEDs; (**c**) normalized far-field distributions of UV LEDs.

The optical and electrical test results are demonstrated in Figure 9. The IV characteristics and series resistances are shown in Figure 9a. The operating voltages at 20 mA were 5.1, 3.85 and 3.85 V for DBR-LED without SPL, DBR-LED with SPL and ST-LED, respectively. The series resistances for the three LEDs were 50, 26.3 and 25 Ω, respectively. These results indicated that the damaged n-AlGaN layer of DBR-LED without SPL had a higher series resistance and would lead to a relatively high working voltage, as reported by other researchers [17,20]. When applied in practical devices, the high voltage would result in low current injection efficiency and heat accumulation, and hence, would affect the device reliability. However, the two-step etching method we proposed could perfectly solve this problem. The light output power and external quantum efficiency (EQE) versus current curves are shown in Figure 9b. The curves of DBR-LED without SPL were not shown in this figure because the luminescence was so weak that it could not be detected due to the damage of the active region by EC etching. The peak values of EQE were 1.97% at 40 mA and 3.04% at 45 mA for ST-LED and DBR-LED, respectively. The output power was 4.55 and 7.54 mW at 100 mA for ST-LED and DBR-LED, respectively. These results indicated that the maximum of EQE was improved by 54.3% at 45 mA, and the output power was enhanced by 65.7% at 100 mA via the use of nanoporous AlGaN DBR in UV LEDs.

Figure 9c shows the far-field distributions of DBR-LED (with SPL) and ST-LED. The far-field pattern of ST-LED agreed well with the Lambertian body which has a cosine intensity distribution with the angle. However, the far-field distribution of DBR-LED (with SPL) showed a smaller divergence angle (110°, full width at half maximum, FWHM) than that of ST-LED (120°, FWHM). The reason for the concentrated divergence angle was that the reflectivity of DBR depended on the incident angle. When the incident angle increased, the reflectance spectrum would shift to the short wavelength side, and the reflectivity would rapidly decline due to the relatively narrow bandwidth of porous DBR (20 nm) in the experiment. A concentrated energy distribution is more beneficial for the application that requires high optical power density such as UV curing, which also makes the DBR-LEDs more favorable to the practical uses than ST-LEDs.

## 4. Conclusions

In summary, we have fabricated nanoporous AlGaN DBRs with the electrochemical etching method. The etching mechanisms were analyzed and the electric field distribution of nanoporous AlGaN stacks was also simulated by the FDTD method. A peak reflectance of 93.5% at the central wavelength of 365 nm was achieved and it was the highest reflectance below 370 nm of porous AlGaN DBRs which has been reported so far. In order to fabricate the DBR-LED, an innovative two-step etching method with SiO_2_ SPL was proposed to protect the n-AlGaN and active region of UV LEDs from the electrolyte during EC etching. The cross-sectional morphologies of DBR-LED with/without SPL were compared. PL spectra of DBR-LEDs were measured and the resonance effect was observed. FDTD simulations were performed to analyze the electric field distribution of DBR-LED and ST-LED, and the results indicated that the light extraction efficiency of UV LED was enhanced due to the highly-reflective porous DBR. The electrical and optical properties of DBR-LED chips were also analyzed. The EL emission spectra were measured, and the EL intensity of DBR-LED was enhanced compared with the ST-LED. In the IV measurement, the DBR-LED without SPL encountered an increasing operating voltage and series resistance due to the porosification of n-AlGaN layer of UV LED, while the voltage of DBR-LED with SPL remained the same as the un-etched ST-LED. In the PI measurement, the maximal EQE of UV LED was improved by 54.3%, and the output power was enhanced by 65.7% at 100 mA by the use of nanoporous AlGaN DBR. Comprehensively, DBR-LED with SPL showed substantial enhancement of light output power, without any degeneration in electrical properties. In addition, the DBR-LED also had a smaller divergence angle due to the angle-dependent reflectivity of porous AlGaN DBR, and this is more beneficial for application such as UV curing. We believe that by optimizing the thickness and Al mole fraction of the u-AlGaN current-blocking layer and n-AlGaN layer of UV LED, the absorption would be reduced and the output power would be further enhanced. This work has paved the way for the application of electrically-pumped UV LEDs and VCSELs based on nanoporous AlGaN DBRs.
